# Life Insurance and the Medical Profession

**Published:** 1884-10

**Authors:** I. N. Danforth


### ARTICLE III.

### LIFE INSURANCE AND THE MEDICAL PROFESSION. *By* I. N. DANFORTH.

[*Read before the Mississippi Valley Medical Society, Sept.* 26, 1884.]

The relations existing between the business of life insurance and the medical profession are, and must continue to be, most intimate. Not a risk can be safely taken until its vital probabilities have been investigated by a duly qualified physician; not a loss can be paid until the cause or causes of death have been certified by the attending physician. Indeed, I do but state a well-worn fact, when I say that the perpetuity of any life insurance company depends very largely upon the ability and integrity of its medical examiners. The ubiquitous " life insurance solicitor," however agile and persuasive he may be, is helpless in the way of achieving practical results, until his work has received the approval of the medical examiner.

The statistics upon which modern life insurance is based,are the outcome of data furnished by medical men, and the more recent trustworthy and accurate statistical tables have been constructed by physicians. Again, life insurance companies depend upon the medical profession of our day for protection against the ravages of what were formerly uncontrolled epidemics. The companies do this, perhaps, with a sort of thoughtless unconsciousness, and always, so far as I have observed, without anything like a generous acknowledgement of their obligations to our profession; but the fact is no less patent, and the obligations are no less deep. In these days o modern travel, when civilized nations are becoming more and more migratory every year, and epidemic influences are, therefore, becoming more generally diffused, life insurance companies could not conduct their business with anything like safety or certainty but for the safeguards which sanitary science, as developed by intelligent physicians, throws around them. It must be remembered that the dangers from the most deadly epidemic diseases have increased incalculably since the introduction of steam as a locomotive agent. All peoples of all nations travel incessantly; merchandise from all parts of the globe, and from all centers of infection, is constantly pouring upon our shores. Yet, how little thought do life insurance authorities give to epidemic diseases as a specially dangerous factor as regards their business: simply because they trust, with a confidence that has been verified over and over again, to the protecting influences of modern sanitary science, which medical men have laboriously developed.

Again, we are in the midst of one of those cycles of discovery which have characterized the profession of medicine from its earliest history. I allude to the "germ theory of disease," which now occupies so prominent a place in the minds of medical men. What the practical outcome of the theories—or, it may be, discoveries—of Pasteur, Koch, and others, will be, no man can yet prophesy with anything like certainty; but if a larger experience shall demonstrate their truthfulness, the effect upon the duration of human life must be very considerable.

Now, all future experiments which shall be conducted with the view of demonstrating the verity or falsity of the germ theories, must be conducted by medical men, experienced in such investigations. The results may be of vast importance in their practical relations to longevity, to preventible causes of disease, or even to the extermination of diseases which are now the terror of the magnates at the "home office." But these latter gentlemen are perfectly helpless themselves as to all such studies. A whole score of presidents, secretaries, directors, actuaries, and general managers would be put to rout by a single "microbe." As between a lively bacterium and a "successful" solicitor, it would be about an even thing.

Now, this plain, simple statement of facts, shows that the business of life insurance casts upon the medical profession a very weighty responsibility—a responsibility, I may add, which, while it came unsought, has come to stay and must be met accordingly. And the time will come when the life insurance world will recognize in a more adequate and audible manner, the vast benefit it has received from the silent and unrequited labors of the great modern medical investigators.

But the medical profession sustains a still nearer relation to life insurance, in the person of the medical examiner—*by far the most important officer on the roster of the company.* In almost any life insurance office, this last statement would be received with derision and contempt. I can myself picture two or three presidents and twice as many secretaries who would shudder at the claim I make concerning the importance of the medical examiner; but this audience of thoughtful physicians will not only recognize but heartily endorse the truth of what I have stated. A life insurance company can get along very comfortably for a time without a president or secretary; or if either of these functionaries vacate their offices, they can be filled at once and worthily; but no company can do business for a single day without its corps of efficient medical examiners, and the loss of an old and the appointment of a new examiner is always regarded at the central office as a grave and important matter.

I propose to consider briefly the duties of the medical examiner, first to the company under whose commission he acts, and, secondly, to the applicant for the benefits of life insurance.

First: Duties and obligations to the company. The medical examiner should, from the first, regard himself as an officer of the company, and not a mere wage-worker or employé. His position is therefore independent and dignified, and not at all subject to the mere whim or caprice of the local agent. From this standpoint of independent and individual responsibility—and this standpoint alone—should the examiner estimate the extent and importance of his duties to the company. The company has a right to expect and demand, first, that the examiner shall recommend sound lives only. A life insurance company is not a hospital or an eleemosynary institution; insuring men is not a work of charity; it is a matter of strict, stern business. A life insurance company *must* "make money" or die, and there is no halting-place between these two alternatives. The examiner ought to keep this fact in mind, namely, that he is not engaged in a work of charity, but that he is simply "doing business" on strictly business principles. The question as to insuring any individual life is simply this, "Will it pay?" "As a business transaction is it safe and desirable?" Hence, the company has a right to expect and demand that each and every examination shall be carefully and thoroughly made; that all sentimentalism and friendship shall be sunk out of sight, and that the investigation shall be coldly judicial, severely searching, and impartially just. It is frequently the case that a single examination is not sufficient to determine certainly the character of a risk: a common catarrhal cold may exist to-day which will be quite well next week; the pulse may be unduly excited by the novelty of the examination; the applicant may have been exposed to some communicable disease, whereof the incubative period has not yet passed;—these and many other causes may render a second examination necessary, if justice is to be done in the premises. In all such cases, the examiner should patiently repeat his work, recognizing the fact that important interests are at stake which his dictum must settle.

Again, there is a class of risks which ought neither to be recommended or rejected; but they ought to be "laid over" for future consideration. As examples, I may mention applicants who have recently recovered from acute diseases which are likely to leave or to cause local lesions, as rheumatism, gout, and pneumonia; applicants who are exhausted by overwork, physical or mental, but who are likely to have easier times in the near future; applicants who are suddenly seized with a passion for loading themselves with life insurance; applicants who are in the midst of heated political campaigns and their accruing dangers; applicants who have recently been reclaimed from intemperance and other forms of vice;—such applicants ought to be suspended for an indefinite term, until their probable permanent status can be more certainly known. Practical experience will enlarge this latter list considerably. It is the almost daily experience of a busy examiner that some applicant has to be "laid over" until some occult point in his personal or family history can be explained satisfactorily. It must be remembered by the examiner that, although he is investigating the *present* condition of the applicant, it is with reference to what the *future* is likely to develop. It is a matter of little concern to the company what the sanitary status of the insuree is to-day, compared with what his status will be in five or ten years hence. The function of the examiner is prophetic; he is required definitely to express his opinion as to what the future sanitary condition of each applicant is likely to be; and this opinion must be based not alone upon the bald statement of facts elicited by the general interrogatories propounded in the application, but upon the harmonious correlation of these facts with each other, as well as with any collateral facts which may develop themselves, even though they be not embraced in the application. The examiner is, therefore, warranted in seeking information concerning the insuree from any and every source, and he is by no means limited to the list of interrogatories submitted by the company, tortuous and formidable as they have recently come to be.

In the next place, the company has the right, not to demand, indeed, but to expect that sort of aid from the examiner which is covered by the word "influence." Regarding every medical examiner, two things are to be taken for granted: First, that he is a thorough believer in life insurance, not only theoretically but practically. I hold that no physician ought to accept a commision as examiner for life insurance until he has "examined" the thing itself, and become convinced, not only of its utility and beneficence, but of its necessity; and, furthermore, that no man ought to be an officer in an institution in which he has not some pecuniary interest himself. Secondly, that no physician will accept a commission as examiner for a company whose standing and condition he has not first investigated with satisfactory results. These two conditions being fulfilled, it is not too much to expect that the examiner shall cast his influence in favor of life insurance in general, and of his own company in particular. Not that he is to sacrifice the dignities of his profession, and turn insurance solicitor; not that he is to divide his time between visiting his patients and "drumming up risks;" nothing could be worse than that. But it is frequently the case that a few quiet words will confirm a doubtful applicant, or turn the thought of a careless spendthrift towards a policy of insurance. And this is a service which is not only legitimate as regards the company, but beneficial and laudable as regards the citizen; and, moreover, it is in no sense derogatory to the professional dignity of the wisest and most learned physician. I recall with pleasure many instances in which I have been instrumental in inducing poor men with families to procure policies of life insurance; knowing, as I do, that thus some provision has been made for those who might otherwise be left in needy helplessness.

In this connection it ought to be said that no examiner should, under any circumstances, allow himself to have any pecuniary interest in the acceptance of a risk. It is sometimes the case that agents—thoughtlessly or otherwise—propose to "divide commissions" with the examiner, especially if the latter has exerted any influence in the case. This is a very dangerous practice; it is justly as well as generally condemned by the company, and should be frowned upon by every medical examiner. Practically it amounts to offering a bribe to the examiner for passing a given risk. Some years ago, I was sent to a little town in interior Illinois to investigate a case which looked somewhat "crooked" upon its face. An insured party—a man about thirty—died of consumption some six weeks after his examination. Upon investigating the case, I found the following facts: the deceased had been a confirmed consumptive for more than two years, and had been treated for this disease by the very man who examined him for life insurance; half a dozen other equally doubtful risks were found, still alive, indeed, but manifesting signs and symptoms which would send the cold chills over the home office; and all these risks had been examined and recommended by this same examiner, and upon his recommendation polices were issued. What was the explanation? Simply this: the local agent and the examiner had "pooled their issues" in the life insurance business; that is, they had agreed to "work" life insurance together, and divide equally the commissions and examination fees, and the result was what might have been expected, as a matter of course. The examiner should in no manner and in no degree compromise his perfect independence of all local influences or dictation.

In the second place, let us look for a few moments at the duties and obligations of the examiner to the applicants for insurance.

Applicants for life insurance have certain *rights* which belong to them, and which cannot be taken from them without injustice; and it happens that they are dependent upon the medical examiners for protection and fair treatment. An applicant ought never to be rejected, except for good and sufficient reasons. It works an injury which is lasting. It practically disbars the injured party from the benefits of life insurance; for a rejection is the mark of a burning iron which is indelible in the eyes of the insurance world. It goes on record at the home office; it becomes the common property of all the companies; and thus it becomes a part of the written history of a man's life. Now, life insurance is every man's privilege, until he is clearly proved to be ineligible; it is a privilege of inestimable value, and one which should not be lightly abridged or destroyed. "In doubtful cases," the company's instructions generally read, "give the company the benefit of the doubt and reject," or words to that effect. This is all very well and very proper; but let the "doubt" be clearly established. Some point in the family history, which the applicant cannot explain without seeking information elsewhere, or the presence of a simple catarrhal cold, or some other trivial and probably transitory objection, ought not to be converted off hand into a "doubt," and therefore a rejection. Let justice be done; let the applicant be given an opportunity to explain his family history, or otherwise "argue his case;" let the bronchial irritation have a few days of grace; let the final decision be postponed a little, and it is not unlikely that a good risk may be saved to the company, and an act of injustice to the applicant be avoided. Some companies require the examiner to classify the subjects he examines, according to their degree of excellence, into first, second and third-class risks. More than fifteen years ago, in reviewing the first edition of my friend Professor Allen's work, on "Medical Examinations for Life Insurance," I expressed my disapproval of this plan in unqualified terms, and subsequent experience and observation has but strengthened my opinion. In the first place, there can be no fixed standard of human physical perfection, and therefore no absolute standard of comparison. Hence, not even the medical director himself can erect a fixed standard of excellence or faultlessness for his subordinates to study as their guide; hence, also, there must in practice be as many standards as there are medical examiners; in other words, every examiner must judge for himself. A very timid and conservative examiner would be likely to commit errors on the side of an extreme conservatism, while a bold and aggressive examiner would commit quite as many errors in the opposite direction. The practical result at the home office would be simply confusion, with corresponding perplexity to the medical director.

But where this system is adopted by the home office it must be followed by examiners. The question then comes up, what is a first-class risk? Of course, if only faultlessly perfect subjects are first-class, they must be few and far between, since it is almost impossible to find a person of adult years who is physically perfect. Again, if anything less than sanitary perfection can be accepted as first-class, at what point shall the line be drawn?—or where shall first-class end and second-class begin? It is evident that no absolute rule can be established, but that each man must in practice erect his own standard, and judge for himself. In my own practice, I am accustomed to rating as first-class all applicants who convince me, upon good and substantial grounds, that they are likely to "live out" their expectatation, and I think this rule is just and fair as regards both applicants and companies.

The medical examiner frequently becomes possessed of information concerning applicants which is confidential in its nature. I need hardly add that all such revelations should be as jealously guarded as though they were made by a patient at a regular consultation. The insuree has the right to demand this, and the law would, without doubt, protect him in that right.

In conclusion, let us consider for a moment the subject of the medical examiner's fees. They are, of course, ridiculously small. Most of the companies pay a uniform fee of three dollars; perhaps half a dozen pay five dollars, while a considerably larger number pay but two dollars. I sometimes hear of the "injustice" of the companies in paying such trivial remuneration for so responsible a service; but I do not so regard it. It is not the business of the companies to crowd larger fees upon us; it is our business to demand them, and that is just what we are not likely to do. Physicians, as a body, are somewhat remarkable for their lack of business acumen, and for their utter want of cohesive force. Whichever way the physician turns, he is victimized by rings and trades-unions; the butcher, the grocer, the plumber, the carpenter, and every other tradesman and artizan is bound and protected by the laws of his guild. But the physician not only fights the trades-unions single-handed, but he also enters into a lively competition with his brother across the way. Now, insurance managers know all this; and so long as we continue to drug the market with medical men to whom time is a burden, and especially so long as we continue to manifest an utter want of union among ourselves, the companies will fix the price to suit themselves, and nobody can blame them.

But the question of a "more perfect union" among physicians—a union which shall adjudicate not only upon matters of ethics, but upon our material and financial interests as well, is one which ought to engage our thoughtful attention.

